# Outpatient Follow-up and Secondary Prevention for Melanoma Patients

**DOI:** 10.3390/cancers2021178

**Published:** 2010-06-07

**Authors:** Ryan G. Gamble, Daniel Jensen, Andrea L. Suarez, Anne H. Hanson, Lauren McLaughlin, Jodi Duke, Robert P. Dellavalle

**Affiliations:** 1 Department of Dermatology, University of Colorado Denver, Aurora, CO, USA; E-Mail: ryan.gamble@ucdenver.edu (R.G.G.); james.jensen@ucdenver.edu (J.D.J.); 2 Kansas City University of Medicine and Biosciences, Kansas City, MO, USA; E-Mail: ahhanson@kcumb.edu; 3 Rocky Vista University College of Osteopathic Medicine, Parker, CO, USA; E-Mail: laurenmcla@gmail.com; 4 School of Pharmacy, University of Colorado, Aurora, CO, USA; E-Mail: jodi.duke@ucdenver.edu; 5 Dermatology Service, Denver Veterans Affairs Medical Center, Denver, CO, USA; 6 Epidemiology Department, Colorado School of Public Health, Aurora, CO, USA

**Keywords:** melanoma, melanoma follow-up, patient management, secondary prevention, melanoma surveillance, melanoma chemoprevention

## Abstract

Health care providers and their patients jointly participate in melanoma prevention, surveillance, diagnosis, and treatment. This paper reviews screening and follow-up strategies for patients who have been diagnosed with melanoma, based on current available evidence, and focuses on methods to assess disease recurrence and second primary occurrence. Secondary prevention, including the roles of behavioral modification and chemoprevention are also reviewed. The role of follow-up dermatologist consultation, with focused physical examinations complemented by dermatoscopy, reflectance confocal microscopy, and/or full-body mapping is discussed. Furthermore, we address the inclusion of routine imaging and laboratory assessment as components of follow-up and monitoring of advanced stage melanoma. The role of physicians in addressing the psychosocial stresses associated with a diagnosis of melanoma is reviewed.

## 1. Introduction

Cutaneous melanoma is one of the most common forms of skin cancer and accounts for the greatest number of skin cancer-related deaths in the United States [1]. Dermatologists and primary care physicians are instrumental in screening and treating at-risk patients for suspicious lesions and initiating multidisciplinary follow-up care for melanoma patients following histopathologic confirmation of the disease. Fortunately, most melanoma patients have thin lesions and are cured with primary lesion excision. Improvements in early diagnosis and more frequent diagnosis of lower-risk patients (*i.e*., those with <1 mm of tumor thickness) have led to increased survival. As a consequence, dermatologists and other physicians are increasingly faced with decisions regarding the long term care and surveillance of cutaneous melanoma patients.

## 2. Secondary Prevention of Melanoma

### 2.1. Behavioral Methods of Prevention

Numerous studies have been conducted on sun-avoidant behaviors and sun protection practices in patients who have already been diagnosed with a cancerous skin lesion. Behavior changes are particularly important for patients diagnosed with melanoma, as these patients are at an increased risk for developing subsequent primary melanomas compared to the general public [2]. Novak *et al*. found that the majority of patients diagnosed with a cancerous skin lesion reported an increase in sunscreen use, wore sun protective hats more often, and avoided the direct sun during the midday hours [3]. Women were particularly likely to increase their sun protection behavior. Overall, 87% of melanoma patients reported an increase in sun awareness. This increase in sun awareness has been attributed to frequent consultation with a physician following diagnosis and treatment [2]. 

However, other studies have been unable to report similarly successful behavior change following melanoma diagnosis. One study found that even though patients with a previous diagnosis of melanoma were more likely to perform skin self-examinations and recognize the importance of prompt treatment of suspicious skin lesions, they were not necessarily more knowledgeable about other associated symptoms or more likely to protect themselves from sun exposure [4]. A similar study also found that only 23% of previously diagnosed patients practiced regular sun protection [5]. Sunscreen was always used by 57% of melanoma patients compared to 28% to 32% use by the general public. However, the rates of sun exposure in cancer survivors did not differ from those in the general public.

These mixed outcomes illustrate the limited value that individual physician-directed patient education may have in altering patients’ sun protection efforts, and that the utilization of several effective programs may be needed in order to achieve behavioral change. While it is important that dermatologists stress the importance of sun protection to melanoma patients, primary care providers can serve a key role by providing advice and referring patients with risk factors [2].

Clinical recommendations after melanoma diagnosis currently include multiple follow-up visits which involve patient education and self examinations of the skin and lymph nodes. A more directed patient education program may also increase the likelihood of patients’ compliance with sun protection. There is evidence that melanoma recurrence and second primary diagnoses are usually found by the patient; however, skin self-examinations may require the help of family members in order to see parts of the body that are less easily observed by oneself [5]. This provides an optimal opportunity for physicians to include family members in melanoma prevention efforts–an important intervention because of the increased risk for melanoma in first-degree relatives of patients with melanoma. Unfortunately, this opportunity is generally overlooked. One study found that only half of first-degree relatives have their skin examined by a physician following the diagnosis of melanoma in a family member [6]. 

### 2.2. Melanoma Chemoprevention

Ideally, melanoma chemopreventive agents taken as prophylaxis would reduce melanoma recurrence, incidence and mortality [7]. While evidence is insufficient to recommend their routine use, candidate agents reviewed here target cholesterol biosynthesis, inflammatory and immune mediators, antioxidants, and cell signaling pathways implicated in cellular transformation. Evidence for nutritional chemoprevention via diet, micronutrients, and dietary supplements is also reviewed.

#### 2.2.1. Statins, Fibrates, and Apomine

Statins and fibrates are anti-lipidemics which lower cholesterol by different mechanisms, and both drugs display antitumor activity in a variety of experimental cancer models [8,9]. While early trials reported significantly fewer melanomas in patients taking fibrates [10,11,12], meta-analyses do not reveal these reductions to be of statistical significance [13,14]. Statins have antiproliferative, proapoptotic, anti-invasive, and radiosensitive effects [15]. However, they may alternatively promote cancer cell growth through their angiogenic effects [16,17]. Apomine is a novel antineoplastic agent that inhibits the mevalonate/isoprenoid pathway of cholesterol synthesis, inhibits cancer cell growth, and induces tumor cell line apoptosis [18,19,20,21]. Researchers recently developed a topical formulation of apomine and demonstrated a statistically significant decrease in tumor incidence in mouse melanoma models [22].

#### 2.2.2. Anti-inflammatory Agents

Overexpression of cyclooxygenase-2 (COX-2) and increased prostaglandin biosynthesis are defining features of several malignancies, and correlate with carcinogenesis [23,24,25,26,27]. The SKICAP-AK trial revealed that NSAID use of short duration was more protective against non-melanoma skin cancers than longer duration of use [28]. A few epidemiological studies have examined the association of NSAIDs with melanoma risk and have found conflicting results [29,30,31,32] A recent case controlled study examining the association between statins and NSAID use and melanoma, demonstrated that control subjects were more likely than melanoma subjects to have reported NSAID or aspirin use for 5 years [33]. While NSAIDs have yet to demonstrate sufficient evidence to be recommended for melanoma chemoprevention, their potential role in melanoma may be directed towards adjuvant treatment of metastases rather than prevention [34]. 

#### 2.2.3. Anti-oxidants

Induction of reactive oxygen species (ROS) in the skin by ultraviolet (UV) radiation is damaging to intracellular organelles, depletes the critical antioxidant glutathione (GSH), and ultimately promotes oncogenic mutations via oxidative DNA damage [35,36,37,38]. *N*-acetylcysteine (NAC), an orally bioavailable antioxidant, replenishes the pool of available GSH [39]. Topical formulations have the potential to decrease UV-mediated GSH depletion and ROS formation [40], and recent human studies support the utility of NAC in pre-UV exposure prophylaxis [41].

#### 2.2.4. Anti-proliferatives

Perillyl alcohol (POH) is a naturally occurring chemical that can slow tumor cell growth by suppressing transcription factor-mediated cell proliferation and transformation [42]. A recent phase IIa study examined reversal of actinic damage following POH *vs.* placebo in patients with sun-damaged skin, and showed that histopathologic score was reduced with low dose POH and that abnormal nuclei were significantly reduced with high dose POH. These compelling results warrant larger, well-controlled studies of POH as a chemopreventive agent as well as efforts to improve dermal penetration and bioavailability of POH-based therapeutics.

#### 2.2.5. Diet, Micronutrients and Nutritional Supplements

Diet, micronutrients, and other nutritional supplements may also play a role in melanoma chemoprevention [43]. Vitamins C [44], D [45,46,47,48], and E [49,50,51,52,53,54,55] each have varying degrees of evidence supporting their use as chemopreventive agents. The same is true with other dietary supplements such as green tea polyphenols [56,57,58,59,60,61], selenium [62,63,64,65], curcumin [66], and lycopene [67,68,69]. While there are many *in vitro* and animal studies that indicate a possible benefit in melanoma prevention, human studies are generally lacking and do not suggest a clear clinical recommendation that physicians should pass on to their patients.

## 3. Diagnostic Follow-up of the Melanoma Patient

### 3.1. Dermatoscopy

Dermatoscopy, also referred to as epiluminescence microscopy or dermoscopy, is currently the most effective clinical modality for diagnosing and screening for melanoma. Essentially skin surface microscopy, this technique allows inspection of skin lesions without obstruction from skin surface reflections. An invaluable tool for monitoring clinically atypical nevi and identifying new primary lesions in melanoma patients, dermatoscopy also increases melanoma diagnostic sensitivity from 60% by naked-eye exam to 90% in experienced hands [70]. Randomized trials have shown up to a 42% reduction in biopsy referral with dermatoscopy compared to control groups [71]. When clinicians are adequately trained in its use, the application of dermatoscopy as a diagnostic tool reduces patient harm and distress and helps eliminate the extraneous cost associated with benign lesion excision. 

When following patients with metastatic melanoma of unknown origin, dermatoscopy may identify key features, including linear-irregular vasculature, scar-like depigmentation, remnants of pigmentation, and pink coloration of the background, assisting the clinician in identifying regressing primary lesions [72]. Furthermore, winding and polymorphic atypical vessels, pigmentary halos, and peripheral grey spots are highly suggestive of cutaneous melanoma metastasis and warrant prompt work-up when examining a patient with previous melanoma [73]. Visualization of these features using dermatoscopy may allow the clinician to more accurately narrow the field of possible lesions responsible for confirmed metastasis with unknown primary lesion, although in most cases, no primary melanoma can be identified [74,75,76].

Patients with a prior diagnosis of melanoma are at higher risk for subsequent melanoma, suggesting the need for a lower threshold to proceed to biopsy of suspicious melanocytic nevi. However, even in high risk patients, such as those with atypical moles or a history of melanoma, lesions that have evolved between successive dermatoscopic examinations are most likely to be dysplastic nevi [77]. In one study, 196 high risk patients with melanocytic nevi were followed for an average 25 months with dermatoscopy, resulting in a ratio of thirty-three lesions excised to two melanomas identified [78]. In another study, 297 high-risk patients were followed for a median period of 22 months, and there was a ratio of 64 dysplastic nevi to one melanoma biopsied due to change on repeat dermatoscopy [77]. Additional biopsies revealed 4 melanomas that arose in skin not previously photographed. The fact that many melanomas arise in previously normal skin limits the sensitivity of dermatoscopic monitoring in high risk populations. 

### 3.2. Reflectance Confocal Microscopy

Reflectance confocal microscopy (RCM) allows for non-invasive evaluation of tissue underlying dermatoscopic structures with cellular-level resolution [70]. Tissue can be viewed in thin horizontal sections from the stratum corneum through the epidermal layers into the superficial dermis. This technique works optimally for light-colored lesions (65% improved specificity), while dermatoscopy seems to be ideal for evaluation of darker lesions [79]. RCM compliments dermatoscopy and allows evaluation of “grey zone” lesions and light-colored or amelanotic lesions not easily evaluated by dermatoscopy (specificity of 39% *vs.* 84%, respectively) [70]. Overall, RCM has superior specificity (68% *vs.* 32%) and similar sensitivity (91% *vs.* 95%) to dermatoscopy in diagnosing melanoma [79]. RCM is especially effective in confirming all non-biopsied control lesions as benign, thus preventing unnecessary biopsies and subsequent patient anxiety [79]. RCM is also a helpful method for monitoring the response of *in situ* residual disease to topical therapies [80]. A limitation of RCM is the significant false-positive rate of identifying Spitz nevi as melanoma. In one study, 56% of Spitz nevi were misclassified as melanoma using RCM [81]. Since there is also uncertainty in the histological differentiation of Spitz nevi from melanoma, a cautious approach should be taken for young patients with multiple Spitz nevi [82]. A false-negative rate of 12% with dermatoscopy compared with a false-negative rate of 9% with RCM shows that the solitary use of any one technique is not ideal. However, the high cost of obtaining the required equipment and amount of time needed to image each lesion limit the availability and use of this modality [83]. Overall, with the two modalities combined, sensitivity increases to 98% while specificity decreases to 23% [79]. For following up high-risk melanoma patients it is advantageous to use both dermatoscopy and RCM, when available, optimizing the chances of identifying a recurrence or second primary lesion. 

### 3.3. Full Body Mole Mapping

Full body mole mapping is a cost-effective and necessary part of routine clinic follow-up for melanoma patients. It is useful for post-excisional monitoring, following suspicious melanocytic lesions, and assessing the treatment response of various dermatologic conditions such as eczema and psoriasis. Since new or changing pigmented lesions and rapid rate of growth are sensitive predictors for melanoma, full body mole mapping can be a powerful tool in the clinical follow-up of melanoma patients [84]. With improving resolution and clarity of close-up images, as well as the ability to archive increasing data within medical record systems, serial imaging of pigmented lesions is likely to become a component of standard clinic follow-up care. The technology is already broadly used, with larger whole body-photo being subdivided with links to the detailed close-up images. Some form of whole body imaging is utilized by 63% of American dermatology residency programs in the management of patients at risk for developing melanoma [85]. 

Sequential imaging increases the likelihood that thin, curable melanoma lesions are identified early in their course, thus reducing morbidity and mortality [86]. In a 2009 study comparing a self- or physician-referred patient cohort with a serial imaging cohort using an automated, low-cost full body scan device, the Breslow depth of melanomas in the non-imaged groups were statistically greater than the Breslow depth of melanomas from the serial scanning cohort [86]. Another report shows improvement in diagnostic accuracy of 13.5% and significant increase in sensitivity using a similar, though non-automated, method [87]. Whole body imaging may be especially useful for tumors with intermediate to high risk of recurrence (lesions > 1 mm depth), serving as a baseline reference for ongoing evaluation [88]. Several studies have shown that approximately one third of prospectively diagnosed melanomas were recognized as a result of change from baseline photographs [89]. Baseline whole body digital photography has also been shown to improve patient self-skin monitoring in patients at high risk for melanoma, increasing self skin examination by over 51% when patients were given books or storage disks with copies of baseline images for comparison [89,90,91]. Whole body imaging has shown that new or changing nevi in patients over the age of 50 are more likely to be melanomas–30% when compared to <1% of new or changing lesions in younger patients [92]. This is consistent with the natural history of benign nevi, which rarely form and often regress after age 50. In one study, the mean number of biopsies performed on patients tracked with total body digital photography *vs.* those who were not was similar, though traditional risk factors still had the strongest influence on decision to biopsy [93]. 

Full body mole mapping is an area of opportunity for improved standardization of management of suspicious melanocytic lesions. One study proposed a set of fifteen poses, chosen for patient comfort and technical feasibility, and the authors suggested that a standard template and quality standards for the images would be advantageous for image comparison in the dermatologic community as well as the development of archival, commercially available software [94]. Serial digital imaging allows for the precise mapping of cutaneous lesions in melanoma patients over time, giving dermatologists another valuable tool in screening for recurrence in melanoma patients.

**Table 1:** Summary of modalities for routine follow-up of melanoma patients.

Modality	Advantages and Disadvantages
Imaging: Chest X-ray, abdominal sonogram, CT, PET	- Low sensitivity, high false positive rates unless Stage III or higher [97] - 50% of follow-up costs [128]
Labs: Liver chemistries, LDH	- Useful in Stage IV disease [95]
Lymph node sonography	- Higher sensitivity than palpation [118,119] - May confer survival benefit [120] - Reveals 13% of relapses [97] - Comprises 24% of follow-up costs [128]
Routine History and full body exam	- Detects >50% of recurrences - Non-invasive - Associated with thinner 2^nd^ primary melanomas [129]
Physical exam and lymph node ultrasound combined	- Detects majority of patients with macroscopic lymph node metastasis - Only 10% of histologically tumor positive sentinel nodes are macroscopically detectable [130]
Patient self-monitoring	- Detects up to three-quarters of reported recurrences [131,132,133,134] - With proper education and/or images, could decrease frequency of follow-up visits [133]
Sequential body-mapping, utilizing digital photography	- Increases detection of thin lesions [86] - Decreased mean Breslow depth at diagnosis [86] - Increased diagnostic accuracy of 13.5%, increased sensitivity, increased specificity depending on dermatologist experience with modality [87]

### 3.4. Follow-up Schedules for Melanoma Patients

Initial tumor parameters of the melanoma lesion determine the frequency of follow-up, with recommendations for follow-up intervals taking into account early detection of a second primary melanoma or of recurrence. In patients with a diagnosis of melanoma, the lifetime risk of developing a second primary melanoma may be as high as 10% [95], and current National Comprehensive Cancer Network (NCCN) Guidelines recommend at least annual skin examinations for life for every melanoma patient [96]. More frequent follow-up examinations are required in patients with more advanced disease, as recurrences are detected in 1.5% of patients with stage I, 18.0% with stage II, and 68.6% with stage III disease [97]. For Stage IA–IIA, NCCN guidelines recommend follow-up visits every 3 to 12 months, for five years, and for Stage IIB–IV, follow-up visits every 3–6 months for the first two years, and every 3 to 12 months for the next three years are recommended. A personal history of prior melanoma or dysplastic nevi, the presence of multiple clinically atypical nevi, a family history of melanoma, and high patient anxiety may prompt the more frequent end of the recommended follow-up schedules. The risk of disease recurrence is highest in the first five years after initial diagnosis. In a retrospective study of 373 patients, the median time interval between initial visit and diagnosis of recurrence was 22 months for stage I, 13.2 months for stage II, and 10.6 months for stage III, with a range of 2.3 to 53.8 months for stage III lesions [95].

### 3.5. Serum Markers

Multiple studies have demonstrated that history and physical exam, with tests directed at symptoms and signs, detect more recurrences than any routine study in patients with Stage I–III melanoma [98,99,100]. While use of surveillance blood tests, such as complete blood count (CBC) and lactate dehydrogenase (LDH) to monitor for disease recurrence in asymptomatic patients with Stage I–III melanoma is common in clinical practice, the clinical utility of these tests has never been well established. New NCCN guidelines recommend against performing any routine surveillance blood tests for these patients [96]. Evidence for these recommendations comes from several studies. In a retrospective analysis, Weiss *et al*. analyzed follow-up data on a group of 261 patients with melanomas greater than 1.7 mm in thickness, some of whom had nodal spread. In the 145 patients who developed recurrences, the recurrence was detected by symptoms in 68%, by physical exam in 26% and never solely by CBC or blood chemistry panel [100]. Mooney *et al*. analyzed data from 1,004 patients with stage I and II melanoma. There were 174 recurrences, with information on the method of first detection available for 154 of these. In this group, recurrence was detected by symptoms in 17%, by physical exam in 72% and never by CBC or liver function tests [99]. In a large prospective analysis of melanoma follow-up strategies of 2,008 patients with Stages I–IV melanoma, history and physical examination detected 47% of recurrences, chest radiographs detected 5.5% of recurrences and an elevated LDH was the first signal of metastasis in only three (0.1%) of these patients [98].

Recently, Leiter *et al*. examined the cost-effectiveness of various follow-up strategies in a subset of 1,969 patients with Stages I–III melanoma from the above study [97]. Overall, blood tests (complete blood count, LDH and alkaline phosphatase) had an average cost per detection of recurrence of $46,909 (this and all costs reported subsequently are based on Medicare reimbursement rates). For chest radiography, the cost per detection was $5,433 and for physical exam it was $733. Due to the cost ineffectiveness of the blood tests and chest radiographs, these authors also recommended a follow-up strategy that does not include routine blood tests and that includes chest radiographs only for patients with stage III melanoma.

While routine blood testing is not cost effective for Stage I–III melanoma, newer serum markers for melanoma have been proposed, including melanoma inhibitory activity (MIA), tyrosinase, 5-S-cysteinyldopa and serum protein S-100B [101]. Of these, MIA and S-100B have been the best studied [102,103,104]. In clinically disease-free Stage II and III patients, S-100B and MIA were found to have a sensitivity of 29% and 22%, and a diagnostic accuracy of 84% and 86%, respectively, for detecting new metastasis [105]. Some evidence indicates these markers may be more useful in high risk patients [106,107]. Since S-100B is best at detecting distant metastasis, elevated levels may warrant further investigation with a modality such as FDG-PET/CT, discussed below. Further study is needed to assess the utility of S100B and MIA as well as the frequency with which these tests should be performed in clinically disease free melanoma patients. 

### 3.6. Imaging

Melanoma commonly metastasizes to the regional lymph nodes, lung and brain, but any organ can be affected [109]. In the follow-up care of melanoma patients, imaging, such as ultrasound, CT, PET and PET/CT should mostly be used to investigate suspicious findings on a history and physical. A low index of suspicion should guide the decision to order symptom directed imaging. NCCN guidelines recommend no surveillance imaging for Stage IA–IIA patients and considering periodic surveillance chest radiograph, CT and/or PET/CT, and annual surveillance brain MRI in Stage IIB–IV patients with no clinical evidence of disease [96]. With all imaging modalities, the risk of increased patient anxiety and undergoing unnecessary, costly and potentially harmful biopsy procedures must be weighed against the possibility of detecting recurrent disease before it is clinically evident and when surgical resection or chemotherapeutic treatment is more likely to be effective. Multiple studies have shown that chest radiographs detect few, if any asymptomatic recurrences in Stage I–IIA patients [98,99,100,109,110]. The flexibility of the recommendations for Stage IIB–IV reflects a lack of quality evidence delineating the best surveillance tests in Stage IIB–IV patients. Chest radiographs are inexpensive and involve little radiation exposure. However, in a study of 994 melanoma patients, early detection of disease recurrence by chest radiograph was not associated with increased survival [111]. While chest CT is more sensitive, this benefit must be weighed against the false-positive rate and radiation exposure, which causes an increased lifetime risk of fatal cancer of 1 in 2,000 [108]. In a study involving 347 asymptomatic stage III melanoma patients, 4.2% of CT scans were true positive and 8.4% were false positive [112]. Though expensive, PET imaging has been shown to be superior to CT for metastatic detection [113] and PET/CT has been shown to be superior to PET or CT alone for melanoma staging [114]. The metabolically active brain produces high background activity on PET imaging, and MRI is the preferred imaging modality for the brain [108]. 

Although not a component of NCCN or American Academy of Dermatology guidelines, some European guidelines recommend routine lymph node sonography as an adjunct to physical exam in assessing the regional lymph node basin, to which melanoma often spreads first [96,115,116,117]. In a meta-analysis of 12 studies and 6,642 patients, Bafounta *et al.* showed that lymph node sonography had significantly higher discriminatory power (OR 1,755) than did palpation (OR 21, p = 0.0001) [118]. Machet *et al.* followed a cohort of 373 patients with Stage I and II melanoma and reported palpation and ultrasound to have a sensitivity of 71% and 93%, and a specificity of 99.6% and 97.8%, respectively [119]. The authors noted that earlier detection of metastases only occurred in 7.2% of patients and unnecessary anxiety, unnecessary biopsy or false reassurance occurred in 5.9% of patients. In their large prospective trial of 1,969 patients with stage I-III melanoma, Leiter *et al.* found that lymph node sonography performed annually in stage I, semiannually in stage II and four times yearly in stage III melanoma patients detected 13% of recurrences, more than all other methods except physical exam [97]. Most recurrences detected were in patients with stage II or III disease, a group for whom the cost per recurrence was $9,361. While there was an apparent survival benefit to early detection of metastasis by lymph node sonography, the design of this study could not exclude lead or length time bias, which may have accounted for this result [120]. Further studies should assess the impact of routine lymph node sonography on survival via randomized trials.

In high risk melanoma patients, recent studies have investigated the use of PET/CT to detect recurrences in patients with elevated S-100B levels [105,107]. In a retrospective analysis of 47 patients with elevated S-100B, Strobel *et al.* found that PET/CT had a sensitivity for detecting metastasis of 97%, a specificity of 100% and an accuracy of 98% [106]. More recently, in a retrospective analysis of 46 melanoma patients with elevated S-100B levels, Aukema *et al.* found that PET/CT had a sensitivity of 100%, specificity of 83% and accuracy of 91% [107]. Each of these studies involved a small number of patients and they used different cutoff values for S-100B: Strobel *et al.* used 0.20 µg/L whereas Aukema *et al.* used 0.10 µg/L. These results warrant further investigation via multicenter trials to determine the utility of directed PET/CT in high risk melanoma patients with elevated S100B.

## 4. Psychosocial Issues and the Melanoma Patient

Although many melanoma patients are diagnosed early and subsequently cured, numerous psychosocial issues may affect a patient who has been newly diagnosed or is being treated for melanoma. In a 2008, the Institute of Medicine (IOM) made a sobering observation when it noted that “some of the most basic psychological and social issues affecting cancer patients aren’t being adequately addressed” by physicians [121]. Unfortunately, the report shows that patients often report that their health care providers fail to acknowledge their struggles, most commonly when it relates to difficulties with depression and finances. Providers have the responsibility to attempt to orchestrate effective communication with their patient, thus allowing them to evaluate both the physiological and psychological needs of the patient. Poor psychosocial outcomes in the context of cancer diagnosis include female gender [122], young age [123], and being unmarried [124]. Other studies have found that cancer patients (including those with melanoma) who have strong social support have enhanced quality of life and better disease outcomes [125]. Providers can help melanoma patients and survivors by offering psychosocial and psycho-education interventions to their patients [126]. It is important for physicians to address both short-term and long-term psychological needs of their patients in order to minimize both physiological and psychological morbidity [127].

## 5. Conclusion

Close clinical follow-up is the foundation of care for melanoma patients. Secondary prevention of melanoma through sun protection counseling, patient education, and perhaps the use of chemopreventive agents are important aspects for follow-up of the melanoma patient. Thorough history and physical exam detect the most recurrences at the lowest cost, and diagnostic accuracy is bolstered when dermatoscopy, RCM and full body mole mapping are employed. While surveillance imaging and blood tests using newer serum markers may be considered for patients with higher stage melanoma, most blood tests and imaging should only be performed based on abnormalities detected by the history and physical. Finally, clinicians must remember to maintain open communication with patients and offer support in order to effectively address both the patient’s medical and psychosocial struggles.
